# Health Care Utilisation and Out-of-Pocket Expenditure Associated with Back Pain: A Nationally Representative Survey of Australian Women

**DOI:** 10.1371/journal.pone.0083559

**Published:** 2013-12-20

**Authors:** Emma R. Kirby, Alex F. Broom, David W. Sibbritt, Kathryn M. Refshauge, Jon Adams

**Affiliations:** 1 School of Social Science, University of Queensland, Brisbane, Queensland, Australia; 2 Faculty of Health, University of Technology Sydney, Sydney, New South Wales, Australia; 3 Faculty of Health, University of Sydney, Sydney, New South Wales, Australia; University of Louisville, United States of America

## Abstract

**Background:**

Back pain impacts on a significant proportion of the Australian population over the life course and has high prevalence rates among women, particularly in older age. Back pain care is characterised by multiple practitioner and self-prescribed treatment options, and the out-of-pocket costs associated with consultations and self-prescribed treatments have not been examined to date.

**Objective:**

To analyse the extent of health care practitioner consultations and self-prescribed treatment for back pain care among Australian women, and to assess the self-reported costs associated with such usage.

**Methods:**

Survey of 1,310 women (response rate 80.9%) who reported seeking help for back pain from the ‘1946-51 cohort’ of the Australian Longitudinal Study on Women’s Health. Women were asked about their use of health care practitioners and self-prescribed treatments for back pain and the costs associated with such usage.

**Results:**

In the past year 76.4% consulted a complementary and alternative practitioner, 56% an allied health practitioner and 59.2% a GP/medical specialist. Overall, women consulted with, on average, 3.0 (SD = 2.0) different health care practitioners, and had, on average, 12.2 (SD = 9.7) discrete health care practitioner consultations for back pain. Average self-reported out-of-pocket expenditure on practitioners and self-prescribed treatments for back pain care per annum was AU$873.10.

**Conclusions:**

Multiple provider usage for various but distinct purposes (i.e. pain/mobility versus anxiety/stress) points to the need for further research into patient motivations and experiences of back pain care in order to improve and enhance access to and continuity of care. Our results suggest that the cost of back pain care represents a significant burden, and may ultimately limit women’s access to multiple providers. We extrapolate that for Australian working-age women, total out-of-pocket expenditure on back pain care per annum is in excess of AU$1.4billion, thus indicating the prominence of back pain as a major economic, social and health burden.

## Introduction

Back pain is a widespread public health and health services delivery issue in Australia, representing a significant burden in terms of Government, private health insurance and out-of-pocket expenditure [1]–[3]. Amongst Australian women back pain has a prevalence of approximately 77% (experiencing back pain) over the life course [4]–[7]. Back pain is a significant primary healthcare issue given the wide range of available self-prescribed treatments, and of providers offering care and utilised by those with pain. Back pain care is highly pluralistic, with biomedical, allied health and complementary and alternative medicine (CAM) practitioners *each* playing significant roles in the delivery of musculoskeletal care [4], [8], [9]. Biomedical practitioners are identified here as those core to the biomedical model and medical curriculum (general practitioner, neurologist, orthopaedic specialist, rheumatologist, and other medical practitioner); allied health practitioners are identified here as those who are associated with the biomedical model and who traditionally offer services to assist the biomedical profession (nurse, occupational therapist, pharmacist, physiotherapist, other allied health practitioner); CAM practitioners are identified here as those who are not traditionally associated with biomedicine (acupuncturist, aromatherapist, craniosacral therapist, chiropractor, herbalist/naturopath, massage therapist, meditation/yoga practitioner, osteopath, reflexologist, reiki therapist, traditional Chinese medicine practitioner, and other alternative health practitioner). This pluralism also means people are required to access a complex mix of Government subsidies (via Medicare, the Australian national health system), private health insurance rebates (with varying levels of funding for musculoskeletal problems) and also pay out-of-pocket expenses [10]. Multiple provider utilisation and patient-driven ‘integration’ of provider options creates a complex landscape of care that presents consumers/patients with a range of claims around expertise, legitimacy and efficacy [11]. Despite some baseline work having been conducted on practitioner utilisation for back pain in Australia [4], [5], [11]–[13] there is a lack of rigorous, representative data which profiles the care sought by back pain sufferers and the costs involved in such activities.

### Back pain and the burden of illness in Australia

Back pain constitutes the second most common complaint in general practice encounters in Australia [5], [14]. Direct and indirect costs are high with estimates placing musculoskeletal problems as a leading disease burden in Australia [2], [6], [7]. Costs not only impact on the State and pressure health services and practitioners, they have differential impacts on individuals, with those with greater economic resources (e.g. high-end private health insurance) more likely to utilise available discretionary forms of care [15]. The Australian health care system maintains a public/private split of around 70/30 respectively [16]. A broader professional ‘division of labour’ between biomedicine, allied health and CAM also shapes access to, and opportunities for, care.

### Providers and competing forms of care

There are a wide range of health care providers who currently offer treatment for those suffering from back pain and this area of illness and disability remains a relative ‘success story’ (in terms of practitioner usage) for the CAM community [10]. Chiropractic is one of the most utilised forms of CAM internationally and its primary focus remains musculoskeletal problems [3]. Moreover, massage therapy and acupuncture, also popular forms of CAM, maintain a focus on musculoskeletal problems [3], [9], [13]. Add this to the relatively few options GPs have to offer for chronic back pain other than pain relief, advice and referral to physiotherapy and other health care providers, and CAM (along with allied health) are key players in back pain care.

Allied health, in particular physiotherapy, is a central stakeholder in conventional approaches to back pain care and the links between general practice and physiotherapy are well established [17], representing the primary biomedical ‘shared care’ alternative. While increasingly GPs do coordinate care with (selected) CAM practitioners [18], research illustrates that scepticism toward CAM and lack of knowledge of CAM practices means that Australian GPs have limited links and referral relationships with many CAM practitioners [19]. Given the use of some CAM and/or allied health practitioners without referral, the issue of cost (and out of pocket expenses from self-initiated consultation) and practitioner engagement becomes even more important for understanding back pain care as a public health and health services issue.

### Back pain expenditure: Individual cost and health system burden

Given the prevalence of back pain, international studies have noted the significance of back pain care expenditure at an individual and health system level [20]–[27]. While general consensus exists as to the increasing financial burden of back pain in developed countries, few studies have investigated the actual out-of-pocket expenditure for individual back pain sufferers. Rather, studies have focused on the broader cost of illness [20], [23], [24], [27], direct and indirect health system costs/burden [21], [22], patterns of expenditure for back pain care [25], [26] and population expenditure on specific practitioners/treatments [28]. As a result, calculations of economic expenditure for both individuals and health systems more broadly are highly variable [20]. To address these gaps in knowledge, this study aimed to uncover and profile health care utilisation for back pain care, and the actual out-of-pocket expenditure for a nationally representative sample of older Australian women.

## Methods

### Sample

This paper reports on a sub-study of the Australian Longitudinal Study on Women's Health (ALSWH). ALSWH was designed to investigate multiple factors affecting the health and well-being of women over a 20-year period. In 1996, women in three age groups (18–23, 45–50, 70–75 years) were randomly selected from the national Medicare database and invited by mail to participate. The respondents have been shown to be broadly representative of the national population of women in the target age groups [29]. The focus of this study is women from the ‘1946-51’ cohort. At the most recent ALSWH survey (survey 6, conducted in 2010) 10,011 women responded, representing 71.0% of the original 14,099 women recruited in 1996. The sub-study survey of this cohort occurred in 2011/2012 when the women were aged 59–64 years. For this sub-study 1,851 women who had indicated in survey 6 (2010) that they had sought help for their back pain were mailed a questionnaire and of these women 1,310 (80.9%) returned completed sub-study questionnaires.

### Ethics statement

Ethical approval was gained from the Human Ethics Committee at the University of Queensland and the University of Newcastle, Australia, and written informed consent was provided by all participants.

### Demographic characteristics

Postcode of residence at the time of the baseline survey was used to classify area of residence as urban or non-urban. Women were asked about their current marital status and the highest educational qualification they had completed.

### Health care utilisation

The women were provided with a list of 5 biomedical practitioners (i.e. general practitioner, orthopaedic specialist, neurologist, rheumatologist, and other medical practitioner) and 5 allied health practitioners (i.e. physiotherapist, occupational therapist, nurse, pharmacist, other allied health practitioner) and asked to indicate if they consulted any of them for back pain during the previous 12 months. The women were also provide with a list of 12 CAM practitioners (i.e. acupuncturist, aromatherapist, craniosacral therapist, chiropractor, herbalist/naturopath, massage therapist, meditation/yoga practitioner, osteopath, reflexologist, reiki therapist, traditional Chinese medicine practitioner, and other alternative health practitioner) and asked to indicate if they consulted any of them for back pain during the previous 12 months. With regard to all of these health care practitioner consultations, the women were asked how much it cost (i.e. out-of-pocket expense) to consult with them. The women were also asked to indicate if they had taken self-prescribed treatments for their back pain in the previous 12 months. The list of treatments included herbal medicines, painkillers (e.g. Panadol, Nurofen), vitamins/minerals (e.g. magnesium), supplements (e.g. glucosamine, fish oils), meditation or yoga, aromatherapy oils, Chinese medicine, self prayer, and other alternative treatments (participant specified). With regard to all these self-prescribed treatments, the women were asked how much it cost to purchase them.

### Health status

The women were asked to indicate the length of time they had experienced back pain and how frequently they experienced the back pain, in the previous 12 months. They were also asked to rate out of 10 (where 0  =  no pain and 10  =  worst possible pain), the intensity of their *typical* back pain, the intensity of their *worst* back pain, the intensity of their back pain at its *best*, and the level at which their back pain was an *acceptable* level of pain, in the previous 12 months. The women were asked to indicate the reasons for consulting with a range of health care practitioners, including pain relief, to improve mobility, to improve function, relaxation/stress relief, general wellbeing. They were also asked to indicate, from a list of 15 the symptoms and conditions related to their back pain, that they sought help for (e.g. headaches/migraines, back pain, neck pain, sleeping problems) and the health care practitioner that they consulted.

### Statistical analyses

Comparisons between continuous and categorical variables were made using Student’s t-test or analysis of variance (ANOVA), where appropriate. All analyses were conducted using the statistical software Stata, version 11.

## Results

The sample consisted of 1310 women, 90% of whom resided in an urban area and 10% in a rural area. The majority (75%) of the women were married or in a de facto relationship, with 21% separated, divorced or widowed, and 4% single. A university degree was attained by 20% of the women, while 21% gained a diploma or certificate, 45% a high school education only, with 14% having no formal education. The women had had back pain for, on average, 20.4 (SD = 13.1) years. Back pain was experienced continuously for 16.2% of women, 39.5% regularly, 40.5% intermittently, and 3.8% rarely. In the past 12 months, women rated (out of 10) the intensity of their *typical* back pain at 5.3 (SD = 2.0), their *worst* back pain at 7.2 (SD = 2.2), and their *best* back pain at 2.4 (SD = 2.2). The women also identified a rating of 3.1 (out of 10) to be an *acceptable* level of back pain.

### Consultations with practitioners and out-of-pocket expense

A total of 1,001 (76.4%) women consulted with a CAM practitioner for their back pain in the past year, 733 (56.0%) women consulted with an allied health practitioner for their back pain, and 775 (59.2%) of the women consulted with a GP/medical specialist for their back pain. Table 1 shows the number of different practitioners consulted, number of consultations, and cost. In the previous 12 months, women consulted with, on average, 1.5 (SD = 1.3) different CAM practitioners, and had, on average, 6.8 (SD = 6.7) CAM consultations. The average out-of-pocket expense of their CAM practitioner consultations was $329.7 (SD = 379.9). In the previous 12 months, women consulted with, on average, 0.7 (SD = 0.8) different allied health practitioners, and had, on average, 2.9 (SD = 4.0) allied health practitioner consultations.

**Table 1:** Number of different practitioner consultations, number of consultations and cost of consultations for alternative health practitioners, allied health practitioners, and GP/medical specialists.

	CAM	Allied Health	GP/Specialist	All Practitioners
Characteristics of Consultations	Practitioners	Practitioners		
	Mean (SD)	Mean (SD)	Mean (SD)	Mean (SD)
**Number of different practitioners**	1.5 (1.3)	0.7 (0.8)	0.8 (0.8)	3.0 (2.0)
**Number of consultations**	6.8 (6.7)	2.9 (4.0)	2.5 (3.3)	12.2 (9.7)
**Cost of consultations**	$329.7 (379.9)	$147.5 (257.9)	$126.8 (246.8)	$604.0 (619.8)

The average out-of-pocket expense of their allied health practitioner consultations was $147.5 (SD = 257.9). In the previous 12 months, women consulted with, on average, 0.8 (SD = 0.8) different GP(s)/specialist(s), and had, on average, 2.5 (SD = 3.3) GP/specialist consultations. The average out-of-pocket expense of their GP/medical specialist consultations was $126.8 (SD = 246.8). Overall, women consulted with, on average, 3.0 (SD = 2.0) different health care practitioners, and had, on average, 12.2 (SD = 9.7) health care practitioner consultations. The average out-of-pocket expense of their health care practitioner consultations was $604.0 (SD = 619.8). In addition to health care practitioner consultations, women also used self-prescribed treatments for their back pain. On average, women used 2.5 (SD = 1.5) different self-prescribed treatments, with an average of 16.5 (SD = 10.9) self-prescribed treatments. The average cost of the self-prescribed treatments was $269.2 (SD = 290.1) (data not shown).

### Total cost of consultations and self-prescribed treatments

In total, women spent an average AU$873.1 (SD = 787.7) on consultations and self-prescribed treatments per annum. Private health insurance in Australia constitutes a tax offset. For this reason, we do not include the economic cost of purchasing private health cover in our calculations of out-of-pocket expense. 68.5% of participants reported having private health insurance, and for those who had a private health insurance policy, expenditure on practitioners for back pain care was greater than for those who did not ($667.5 and $481.3 respectively, data not shown). The average costs of self-prescribed treatments for those who did and did not have private health insurance were $281.0 (SD = 305.9) and $246.3 (SD = 255.7) respectively (data not shown). Thus, the overall average cost per annum for those with private health insurance was $948.5, compared to $727.6 for those who did not (data not shown).

In Australia, there are approximately 750,000 women aged 59–64 [30]. Thus, the total out-of-pocket expenditure for Australian women of this age bracket per annum (previous 12 months) for back pain care is AU$120.5 million. In order to estimate the total out-of-pocket expenditure for working age (15–64 year old) women in Australia, we calculated the weighted proportions of the Australian female population in accordance with rates of seeking help for back pain care from the most recent ALSWH surveys of the 1946-51 cohort (Survey 6, 2010: of which 18.4% sought help for back pain) and the younger cohort (Survey 5, 2009, then aged 31–36: of which 23.0% sought help for back pain; data not shown). In Australia, women aged 15–49 constitute 48.6% (n = 5,394,114) of the total population of women; women aged 50–64 constitute 18.3% (n = 2,031,117) of the total population of women [30]. Extrapolating from these figures, and assuming an average individual out-of-pocket expenditure in line with that of the 1946-51 cohort outlined in this paper (AU$873.10), we calculate the total out-of-pocket expenditure for Australian women aged 15–64 to be approximately AU$1.4 billion per annum.

### Pain characteristics and consultation patterns

Table 2 shows various characteristics of back pain and their associations with health care practitioner consultations. Shorter periods of back pain predicted consultation with a GP/specialist (p = 0.020) whereas longer periods of time predicted consultation with a CAM practitioner (p = 0.003). This suggests that as time goes by, women may extend beyond the more traditional biomedical options to explore CAM.

**Table 2:** The characteristics of back pain and their associations with health care practitioner consultations.

Characteristics of Back Pain	Consulted a CAM Practitioner			Consulted an Allied Health Practitioner			Consulted a GP/Specialist			Used Self-Prescribed Treatment		
	Yes	No	p-value	Yes	No	p-value	Yes	No	p-value	Yes	No	p-value
**Time with back pain (yrs)**			0.003			0.535			0.020			0.418
**Mean**	21.0	18.4		20.6	20.2		19.7	21.5		20.5	19.0	
**SD**	18.4	12.7		13.2	12.9		13.1	13.1		13.0	15.3	
**Typical back pain intensity**			0.914			<0.001			<0.001			<0.001
**Mean**	5.3	5.3		5.7	4.9		5.9	4.4		5.4	3.7	
**SD**	1.8	2.3		1.9	2.0		1.8	1.9		1.9	2.3	
**Worst back pain intensity**			0.001			<0.001			<0.001			<0.001
**Mean**	7.4	6.9		7.7	6.6		8.0	6.1		7.4	5.0	
**SD**	2.1	2.5		1.9	2.4		1.7	2.4		2.1	3.0	
**Best back pain intensity**			0.017			<0.001			<0.001			0.004
**Mean**	2.3	2.6		2.7	1.9		3.0	1.4		2.4	1.7	
**SD**	2.1	2.4		2.2	2.0		2.2	1.7		2.2	2.2	
**Frequency of back pain**												
**Never (%)**	2.4	8.5	<0.001	1.1	7.3	<0.001	0.7	8.5	<0.001	2.6	25.0	<0.001
**Rarely (%)**	40.7	39.6		35.5	46.8		27.5	59.3		40.6	38.9	
**Sometimes (%)**	41.0	34.6		43.1	34.9		47.3	28.1		40.4	23.6	
**Often (%)**	15.9	17.3		20.3	11.0		24.5	4.1		16.4	12.5	

### Reasons for consultations: Biomedicine, allied health and CAM

The reasons considered important in women’s decision to consult with a range of health care practitioners are presented in Table 3. GPs/specialists were the most common practitioner group consulted for pain relief (59.1%), followed by chiropractors (31.3%), physiotherapists (25.5%), and massage therapists (20.5%). Physiotherapists (31.7%), together with chiropractors (30.4%), were the most common practitioner groups consulted for mobility improvement, followed by GPs/specialists (24.0%), and massage therapists (20.6%). Similarly, to improve function, women were more likely to consult with physiotherapists (23.9%) and chiropractors (23.9%), as well as GPs/specialists (20.0%) and massage therapists (16.7%). Massage therapists were the most common practitioner group consulted for relaxation/stress relief. GPs were the most common practitioner group consulted for general wellbeing (26.1%), followed by massage therapists (22.5%) and chiropractors (15.2%).

**Table 3:** The reasons considered important in women’s’ decision to consult with practitioners.

	GP/	Chiro-	Acupunct-	Herbalist/	Physio-	Massage	Osteopath
Reasons for consultation	Specialist	practor	urist	Naturopath	therapist	Therapist	
**Pain relief** (% yes)	59.1	31.3	9.4	3.2	25.5	20.5	7.7
**To improve mobility** (% yes)	24.0	30.4	6.2	2.0	31.7	20.6	6.0
**To improve function** (% yes)	20.0	23.9	5.7	2.7	23.9	16.7	5.8
**Relaxations/ Stress relief** (% yes)	11.1	8.2	3.9	2.6	6.6	22.5	1.9
**General Wellbeing** (% yes)	26.1	15.2	4.4	5.3	9.7	17.1	2.4
**Other** (% yes)	3.6	0.7	0.2	0.2	0.5	0.4	0.3

One point of differentiation was the practitioner groups’ focus on the entirety of symptomatology. That is, whether a particular mode of treatment followed a singular approach, or one focused on the total illness. Table 4 shows the back pain related symptoms and conditions women sought help for and the practitioners they consulted. GPs/specialists were the most common practitioner group consulted for all symptoms and conditions, with the exception of neck pain where they were the second most common practitioner group consulted, behind chiropractors. Chiropractors were the second most common practitioner group consulted for most symptoms and conditions, apart from stiffness, fatigue, instability, muscle spasms, and anxiety/tension, where massage therapists or physiotherapists were the second most common practitioner group consulted.

**Table 4:** The back pain related symptoms and conditions women sought help for and the practitioners consulted.

	GP/	Chiro-	Acupunct-	Herbalist/	Physio-	Massage	Osteopath
Reasons for consultation	Specialist	practor	urist	Naturopath	therapist	Therapist	
**Headaches/Migraines** (% yes)	16.7	9.9	1.7	0.8	5.2	6.0	1.6
**Nausea** (% yes)	9.2	1.4	0.2	0.3	0.4	0.6	0.2
**Back pain** (% yes)	41.0	27.8	6.8	2.9	25.4	22.1	5.3
**Neck pain** (% yes)	20.1	23.4	3.4	1.6	17.0	18.0	4.0
**Leg pain/sciatica** (% yes)	28.9	17.3	3.8	1.5	14.7	14.4	3.4
**Arm pain** (% yes)	13.9	8.0	1.6	0.7	8.0	7.4	1.8
**Pins and needles/numbness** (% yes)	20.2	7.3	1.5	0.7	6.6	4.8	1.6
**Stiffness** (% yes)	15.7	11.5	2.5	0.8	12.0	14.5	2.8
**Fatigue** (% yes)	18.6	2.4	1.3	2.8	1.2	2.9	0.5
**Weakness** (% yes)	11.5	3.4	0.5	0.9	3.3	2.1	0.9
**Depression** (% yes)	18.3	0.8	0.2	1.2	0.2	0.8	0.0
**Sleeping problems** (% yes)	26.8	2.8	1.0	2.3	1.8	2.4	0.5
**Instability** (% yes)	5.2	1.2	0.1	0.2	2.1	0.6	0.3
**Muscle spasm** (% yes)	17.2	7.0	1.6	1.3	8.3	9.2	1.8
**Anxiety/tension** (% yes)	20.3	1.8	0.8	1.7	1.7	4.5	0.2

## Discussion

In this paper we have reported the first national, representative study of women’s back pain care in Australia, focusing on self-reported treatment utilisation and cost amongst the 1946-51 cohort of the Australian Longitudinal Study on Women’s Health. Our analysis reveals a number of significant findings of relevance to health service delivery and clinical practice, including practitioner usage trends, reasons underpinning uptake of services, and the out-of-pocket costs for women and their families. Previously, usage and cost data has been established predominantly through practitioner data and rebate information [19]–[24]. While this covers some aspects of service provision, examining women’s accounts of care (both practitioner delivered and self-prescribed) provides insight into the breadth of engagement and use across the spectrum of providers (biomedicine, allied health and CAM). Such an approach provides much needed data on into women’s management of the multiple available sources of care.

Further reinforcing the prominence of CAM practitioners and practices in the context of back pain care, 76.4% of the women consulted a CAM practitioner in the past year versus 56% for allied health and 59.2% for a GP/specialist [8], [10], [13]. While such widespread usage of CAM for back pain has been previously documented [8], [13], CAM practitioners remain significantly marginalised in terms of Government funding and to some extent even in the context of private health insurance rebates [31]. Thus, this finding illustrates the ongoing paradoxical situation within musculoskeletal care of significant grassroots support amongst back pain sufferers for CAM [4], [5], [10]–[12] *alongside* ongoing structural marginalisation. While this position is increasingly challenged by many CAM practitioner associations, questions of safety and efficacy within the context of manipulation (in particular) continue to limit any shift in existing structural funding and subsidy programs [32].

Regardless of issues related to clinical effectiveness and safety, the results of the current study illustrate a clear economic burden emerging from living with, and seeking help for, chronic back pain. Such costs must be acknowledged and inform the care and referral practices of health practitioners. The out-of-pocket expenditures for back pain care estimated here are large. Given similarities across the female population in terms of consultation patterns and levels of back pain [5],[9],[13], total weighted out-of-pocket expenditure for the working age Australian female population (aged 15–64, 66.9%) [30] is likely to be in excess of $1.4 billion annually. Back pain thus not only represents a major burden in terms of participation in work and family life but also adds economic pressure. While Australian women clearly access multiple providers, this may ultimately be restricted by the substantial costs associated with concurrent practitioner use. As such, economic constraint and existing forms of social marginalisation may be manifest in back pain care and recovery, limit quality of life and capacity to work. It is important that policy makers and health service providers acknowledge these (often) hidden costs and provide support services for women who cannot afford out-of-pocket expenses for maintaining their health and wellbeing.

Although existing work has shown the influences of demographic and socioeconomic characteristics on practitioner utilisation for back pain care [13], little has been explored in relation to the *reasons* underpinning the use of particular practitioners or practices, despite the plurality of available providers [4], [5], [10], [11]. Our results show significant variation in the reasons underpinning help-seeking including: GPs rated highest in ‘*pain management*’; allied health and CAM rated equal highest in relation to ‘*mobility/function*’; and, CAM rated highest in terms of ‘*relaxation/stress*’. Such results suggest a diversified landscape of care with specific strengths and roles across different practitioner groups. Moreover, they highlight the importance of maintaining effective inter-professional as well as patient-clinician communication about *what* people are using and for what *purpose* [10], [33].

There are several limitations to our study. First, our findings may be potentially impacted by the effects of recall-bias, as the health and health care utilisation data is self-reported by the participants. However, the validity and reliability of questionnaire-based instruments, particularly in comparison to medical record assessments for example, has been previously evidenced [34]. A further limitation of our study relates to our extrapolation and estimation of the per annum out-of-pocket expenditure for the overall population of working age Australian women. Rates of help seeking for back pain may be slightly variable outside of the two nationally representative age cohorts from which we have drawn our data. In addition, we acknowledge that extent of practitioner/treatment utilisation, and thus cost, may also vary according to age. We offer the finding of total out-of-pocket expenditure for working age Australian women based on the largest and most recent nationally representative data available. As such, our estimation of overall per annum cost offers the first insight into the huge financial burden for back pain sufferers, which can be augmented by further investigation at a population level. Further research on the influences of demographic and socioeconomic characteristics on out-of-pocket expenditure will also add to understandings of the economic costs for Australian back pain sufferers.
